# Role of MicroRNAs in Renal Parenchymal Diseases—A New Dimension

**DOI:** 10.3390/ijms19061797

**Published:** 2018-06-17

**Authors:** Saeed Kamran Shaffi, David Galas, Alton Etheridge, Christos Argyropoulos

**Affiliations:** 1 Department of Internal Medicine, Division of Nephrology, University of New Mexico, Albuquerque, NM 87131, USA; cargyropoulos@sauld.unm.edu; 2 Pacific Northwest Research Institute, Seattle, WA 98122, USA; dgalas@pnri.org (D.G.); aetheridge@pnri.org (A.E.)

**Keywords:** miRNA, micro RNA, renal parenchymal diseases, miRNA in renal parenchymal Diseases, miRNA detection, miRNA-based therapeutics

## Abstract

Since their discovery in 1993, numerous microRNAs (miRNAs) have been identified in humans and other eukaryotic organisms, and their role as key regulators of gene expression is still being elucidated. It is now known that miRNAs not only play a central role in the processes that ensure normal development and physiology, but they are often dysregulated in various diseases. In this review, we present an overview of the role of miRNAs in normal renal development and physiology, in maladaptive renal repair after injury, and in the pathogenesis of renal parenchymal diseases. In addition, we describe methods used for their detection and their potential as therapeutic targets. Continued research on renal miRNAs will undoubtedly improve our understanding of diseases affecting the kidneys and may also lead to new therapeutic agents.

## 1. Introduction

Ribonucleic Acids (RNAs) are a ubiquitous class of unbranched polymeric molecules that serve as intermediates responsible for decoding genetic information from DNA to ribosomes in the form of messenger RNA (mRNAs), transfer of amino acids to ribosomes by transfer RNA (tRNA), and in protein synthesis itself in the form of ribosomal RNA (rRNA). In 1993, a new class of non-coding RNA molecule, microRNAs (miRNAs), was identified. These small molecules play pivotal roles in cell-growth cycle regulation, differentiation, and survival by modulating mRNA stability and translational efficiency. Over the last two decades, the role of miRNAs in various diseases, as well as their role in maladaptive repair, has been elucidated. In addition, miRNAs have been studied for their potential use in disease diagnosis and prognostication, and as therapeutic targets.

In this review, we describe the role of miRNAs in renal physiology and pathology and their putative roles in various renal parenchymal diseases. We also discuss methods of their measurement as well as various strategies for using miRNAs as therapeutic agents.

## 2. Search Strategy

To identify pertinent studies for this review, we searched Medline for phrases “MicroRNAs” [Mesh] OR “MIRN632 microRNA, human” [Supplementary Concept] AND “Kidney” [Mesh]. We limited our search to papers published in the English language journals from January 2007 to June 2018. We also identified relevant studies from the previously published review papers in this field.

## 3. miRNA Discovery and Biogenesis

Before 1993, it was thought that mRNAs, transcribed from the coding regions of DNA, are translated by ribosomes into proteins with no other non-coding regions of DNA playing a significant role in the processes of protein synthesis or regulation of gene expression. The processes that lead to miRNA biogenesis have been extensively described and we refer readers to comprehensive reviews for details [1,2,3,4]. At the time of writing this review, more than 28,000 miRNAs have been identified, out of which 2588 are human. A central repository of known miRNA sequences has been established (miRBase) [5].

## 4. Role of miRNAs in Renal Development

In mammals, renal development is heralded by aggregation of cells in the mesoderm called the urogenital ridge—a portion of which forms the nephrogenic cord. The nephrogenic cord undergoes changes to form the pronephros which regresses, mesonephros which forms the mesonephric duct, and the metanephros which develops as an outgrowth of cells from the mesonephric duct called the ureteric bud. Nephron progenitor cells surround the ureteric bud and form nephrons. The ureteric bud forms the collecting system of the kidneys. These processes involve a series of well-coordinated steps which require expression and suppression of various proteins that have been shown to be regulated by miRNAs.

To better understand the role played by miRNAs in the developing kidney, researchers conditionally deleted Dicer in specific cell populations in the developing kidney. A mouse conditional deletion of Dicer in the nephron progenitor cells resulted in increased apoptosis with a marked reduction of cells and increased expression of pro-apoptotic Bcl-2-Like Protein 11 (BIM) protein in the embryonic kidney [6]. Nephron number was also substantially reduced. Few nephrons that were present exhibited capillary wall abnormalities. Expression of mmu-miR-10a, mmu-miR-17-5p, and mmu-miR-106b was reduced. Suppression of these miRNAs has been associated with increased pro-apoptotic BIM protein activity [7]. Thus, nephron progenitor cell growth is encouraged by miRNA-mediated inhibition of the apoptotic protein, BIM. Similarly, a mouse conditional deletion of Dicer in the ureteric bud caused disruption of ciliogenesis, resulting in small and cystic kidneys and obstruction of urinary flow leading to hydronephrosis [8].

Although these observations highlight the relevance of miRNA regulation in renal development, the identity of the miRNAs and cell populations involved remains unclear. The first question was addressed by targeted deletion of the mmu-miR-17~92 cluster, which has essential roles in development and has been implicated in cancer. Deletion of this family of miRNAs resulted in the preservation of the nephron progenitor population but impaired their proliferation and thus reduced nephron number. Mice lacking the miR-17~92 cluster developed albuminuria by 6 weeks, and focal podocyte effacement and glomerulosclerosis by 3 months [9]. Ablation of Dicer from maturing renal tubular epithelial cells reduced mmu-miR-200 cluster expression levels and upregulated the polycystic kidney disease 1 (PKD1) gene. Predictably, enhanced PKD1 is associated with inhibition of tubulogenesis and cyst formation [10]. In the same study, it was shown that the PKD1 gene was downregulated by miR-mmu-miR-200b/c using a variety of in vitro approaches. Please note that the miR-200 cluster plays a regulatory role in the epithelial-to-mesenchymal transition (EMT) process which is central to fibrotic pathogenesis, suggesting that developmentally relevant miRNAs may also play important roles in the initiation and progression of kidney disease in the adult life. This concept was validated in another series of experiments, which examined the effects of selective inactivation of Dicer in mouse podocytes early in life [11,12]. This highly specific lesion caused proteinuria and death with renal histology showing foot process effacement, collapsing glomerulopathy, podocyte vacuolization, hypertrophy, and apoptosis. These histological features are also observed in severe forms of the nephrotic syndrome in adult animals and humans (e.g., due to focal segmental glomerulopathy). Specifically inactivating Drosha in podocytes led to collapsing glomerulopathy similar to Dicer knock-out mice [13].

Table 1 summarizes data on the role of miRNAs in renal development. Collectively, these results suggest that the establishment of renal structure and maintenance of kidney architecture is dependent on expression of multiple miRNAs expressed in different compartments within the kidney.

## 5. Role of miRNAs in Renal Physiology

miRNAs play a diverse role in normal renal function, as demonstrated by the elimination of specific miRNAs and/or miRNA-processing enzymes in mouse models. For example, conditional deletion of Dicer in renin-expressing cells in mouse kidneys resulted in a reduced juxtaglomerular cell population, and decreased expression of *Ren1* and *Ren2* genes, leading to decreased renin concentration, hypotension, abnormal renal function, renal vascular abnormalities and strip fibrosis [14]. Conditional deletion of Dicer in podocytes in the prenatal period not only affects normal renal development but also leads to both structural and functional aberrations after nephrogenesis [11]. A major physiological derangement in progressive renal impairment is the inability to fine tune the balance between the excretion of sodium and conservation of potassium. Such alterations underlie the sodium and potassium retention seen in progressive kidney disease in humans. In that regards, it has been shown that specific miRNAs are involved in fluid and electrolyte handling. A mouse model with selective mmu-miR-192-5p knock-out in the proximal convoluted tubule, the site of the fine regulation of sodium balance in the kidney, exhibits upregulation of the Na^+^/K^+^ ATPase β-1 subunit [15]. These animals were unable to increase urine output when fed a high sodium diet [15]. This failure of the adaptive mechanism of sodium natriuresis could contribute to sodium and water retention, which is a common pathophysiological alteration in human kidney disease. microRNAs are also involved in the tight co-regulation of sodium excretion by the kidney in the feed-forward (FF) inhibitory control loops of the with No Lysine kinase system (WNK).

**Figure 1:**
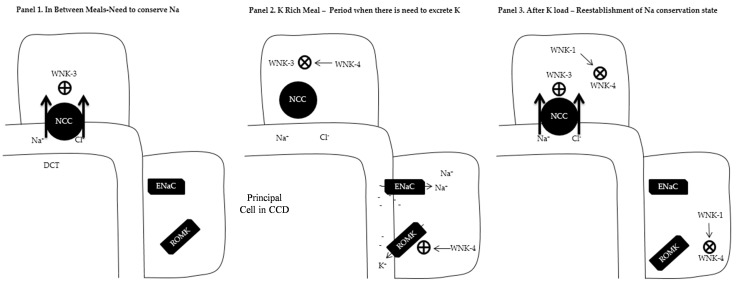
Overview of the with no Lysine Kinase (WNK) system. Abbreviations: NCC: Sodium/Chloride cotransporter; DCT: Distal Convoluted Tubule; CCD: Cortical Collecting Duct; ENaC; Epithelial Sodium Channel; ROMK: Renal Outer Medullary Potassium Channel; ⊕ Increase expression; ⨂ Decrease expression. (**Panel 1**) In between meals when the kidney retains Na^+^ and K^+^. This is mediated by the presence of WNK3 which increases the expression of NCC in the DCT as well as prevents ROMK expression in the CCD. (**Panel 2**) K^+^ rich meal period when there is need to excrete K^+^. Expression of WNK4 causes suppression of WNK3 which leads to diminished presence of NCC in the DCT and increased Na^+^ delivery to CCD. In the presence of aldosterone, ENaCs are expressed in the CCD with electrogenic Na absorption making the lumen negative. WNK4 increases the expression of ROMK in the CCD with the removal of K down the electrical gradient. (**Panel 3**) After K rich meal period. WNK1 antagonizes WNK4 with re-expression to WNK3 phenotype (**Panel 1**).

This system is of emerging importance for understanding the development of systemic, volume-sensitive hypertension. Control of the system of miRNAs exemplifies the integration between FF kinase and epigenetic regulatory loops and thus will be examined at some length here (Figure 1). In the normal state, this system ensures renal switching of roles from inter-meal sodium retention to post-meal sodium (natriuresis) and potassium (kaluresis) excretory states. WNK3 upregulates expression of the NaCl cotransporter (NCC) in the distal convoluted tubule of the nephron resulting in sodium retention. On the other hand, natriuresis is mediated by WNK4, which antagonizes WNK3 and decreases NCC expression. WNK4 also increases the expression of renal outer medullary potassium (ROMK) channels in the distal convoluted tubules, thus promoting kaluresis. WNK1 exerts a major regulatory role in switching between the phenotypes of sodium retention and natriuresis by cleaving WNK4, which in turn removes the antagonism on WNK3 mediated sodium retention. It has been shown that mmu-miR-192-5p negatively regulates WNK1, as sodium depletion, aldosterone infusion, and potassium load led to significant kidney-specific WNK1 mRNA expression and reduction in mmu-miR-192-5p expression [16]. This study, in addition to the miR-192 antagonism results presented previously [15], highlights the potential of miRNAs to serve as context-specific regulators: sodium depletion led to a decreased mmu-miR-192-5p level which was associated with decreased urine output. On the other hand, antagonism of mmu-miR-192-5p by a specific antagomir affected urine output only in the setting of high, but not normal salt intake [15]. Hence a single miRNA (mmu-miR-192-5p) appears to play a major regulatory role in one of the most tightly controlled kinase systems in the kidney. Renal potassium handling may be directly controlled by miRNAs independently of effects on the WNK system. High-potassium diet increased mmu-miR-802-5p transcription in the cortical collecting duct in mice, which in turn decreased expression of caveolin-1, which suppresses ROMK activity [17]. mmu-miR-9-5p and mmu-miR-374-5p suppress claudin-14 which in turn suppresses claudin-16 and 19 paracellular cation channels responsible for Ca absorption in the thick ascending limb of the loop of Henle, a major site of sodium, potassium and calcium exchange in the kidney [18]. Extracellular calcium levels also directly regulate mmu-miR-9-5p and mmu-miR-374-5p levels [18].

Table 2 summarizes key studies investigating the role played by miRNAs in maintaining renal control over sodium and potassium handling. It is evident that miRNAs provide an extra level of complexity and integration of the different systems that maintain the electroneutrality of urine on the one hand while maintaining homeostasis on the other.

## 6. Role of miRNAs in Renal Fibrosis and Maladaptive Repair

Renal fibrosis is the final common pathway of various forms of progressive renal disease. TGF-β signaling plays a central role in renal fibrosis. Renal parenchymal cells synthesize TGF-β1 and its isoforms (β2 and β3). Experimental models and human studies have shown that TGF-β1 is upregulated in diseased and fibrotic kidneys [19]. Various stressors inducing stimuli such as hyperglycemia [20], angiotensin II [21], and reactive oxygen species [22] increase TGF-β1 production. It is then activated and exerts its effects in autocrine and paracrine fashion via Smad-dependent and/or Smad-independent pathways [23]. TGF-β1 initiates Smad2 and Smad3 complex formation with Smad4, leading to its activation, translocation to the nucleus, and ultimately transcription of its targets [23]. It is important to note that Smads2 and 3 can also be activated by mediators other than TGF-β [24]. Noting the role of TGF-β in renal fibrosis, we will now discuss the interplay between TGF-β and various miRNAs.

miRNAs regulate TGF-β activity by modulating expression of various components of the TGF-β signaling pathway. In particular, hsa-miR-744-5p has been shown to post-transcriptionally inhibit expression of the TGF-β1 ligand [25]. Similarly, rno-miR-200a-5p has been shown to repress expression of TGF-β2, which prevents renal fibrogenesis [26].

Conversely, TGF-β signaling also influences miRNA expression. TGF-β administration increases expression of miR-192-5p in human, mouse, and rat tubular epithelial, mouse mesangial, and rat proximal tubular epithelial cells, respectively [27,28,29,30]. Low hsa-miR-192-5p is associated with interstitial fibrosis and tubular atrophy [31]. TGF-β is well known to regulate the expression of the miR-200 family of miRNAs, and administration of TGF-β does indeed lead to decreased expression of the miR-200 family in kidney and rat proximal tubular epithelial cells [26].

The final common pathway of TGF-β signaling is the production of extracellular matrix (ECM) proteins and their deposition into the interstitium. Several lines of evidence demonstrate that this process is under miRNA control. In systemic sclerosis—a disease characterized by widespread fibrosis—expression of the mmu-miR-29 family is decreased [32]. mmu-miR-29a-5p suppresses the expression of collagen type I and III [32]. Furthermore, TGF-β suppressed miR-29a-5p which resulted in feedforward upregulation of TGF-β [32]. A mouse model of bleomycin-induced skin fibrosis was associated with decreased mmu-miR-29-5p, which was reversed by the tyrosine-kinase inhibitor imatinib [32], a potent inhibitor of the TGF-β pathway. The role of miR-29 appears not to be limited to systemic sclerosis since a mouse model of mmu-miR-29-5p inhibition demonstrated protection against salt-induced hypertensive renal sclerosis [33]. There was up-regulation of various genes involved with laying of ECM when mmu-miR-29-5p was silenced in the kidneys of these animals [33]. miR-337 was shown to be involved in diabetic nephrosclerosis [34]. It has recently been shown that miR-337 was upregulated when cultured human and mouse mesangial cells were exposed to high glucose and TGF-β to imitate a diabetic milieu [34]. Fibronectin—a key protein involved in fibrosis—was in fact directly induced by miR-377 [34]. Other animal models have been used to study the role of miRNAs in renal fibrosis: hsa-miRNA-449a/b-5p expression was downregulated in hypoxic fibroblasts [35]. Furthermore, hsa-miRNA-449a/b-5p caused upregulation of profibrotic proteins (serine protease inhibitor protein 1-SERPINE1) [35]. Table 3 summarizes some of the studies that investigated the role played by miRNAs in the pathogenesis and maintenance of renal fibrosis. These experiments show that several profibrotic proteins are under the control of miRNAs.

## 7. miRNAs in Select Renal Parenchymal Diseases

miRNA expression profiles have been studied in many renal parenchymal diseases. Specific miRNA expression signatures have been identified for some diseases in both animal models and human studies. We will briefly review some of these associations since they provide the basis for detecting miRNAs as disease-specific biomarkers and potential therapeutic targets.

### 7.1. Diabetic Nephropathy

miRNAs have been directly implicated in the pathogenesis of diabetic nephropathy. mmu-miR-29c-5p expression—which is associated with podocyte apoptosis—is increased in both the glomeruli and microvascular endothelial cells in a mouse diabetic model [36]. In addition, mmu-miR-29c overexpression promoted activation of the Ras homolog gene family, member A (RhoA)—by suppressing the Sprouty homolog (Spry) 1 gene—which has been shown to play a role in the pathogenesis of diabetic nephropathy [37]. Analysis of kidney biopsy samples from patients with diabetes revealed that hsa-miR-192-5p expression was inversely related to tubulointerstitial fibrosis and directly related to estimated glomerular filtration rate (eGFR) [31].

This association may be causal since the introduction of TGF-β to proximal convoluted tubule cells exposed to high glucose conditions leads to decreased hsa-miR-192-5p expression [31]. Conversely, overexpression of hsa-miR-192-5p ameliorated the TGF-β-mediated fibrosis [31]. Hence, once TGF-β has been activated in high glucose conditions, the decreased expression of hsa-miR-192-5p brought about by TGF-β may further amplify tissue fibrosis.

Several miRNAs may be involved in the expression of the fibrotic renal phenotype. TGF-β increased mmu-miR-216a-5p and collagen type I α1 expression in mouse mesangial models of diabetes [38]. A miRNA circuit has been shown to be directly involved in mediating the autoregulation of TGF-β and the production of ECM [39]. TGF-β induced mmu-miR-192-5p inhibits the expression of the E-box repressors Zeb1/2 which in turn increases the expression of mmu-miR-200b-5p and mmu-miR-200c-5p [39]. These miRNAs further inhibit Zeb1/2 leading to enhanced expression of TGF-β and the ECM components collagen type Iα2 and collagen type IVα1 [39]. Hyperglycemia activates phosphatidylinositol (PI)—3 kinases/Akt pathway leading to cell hypertrophy and increased matrix protein in mouse diabetic models [40]. mmu-miR-21-5p mediates this process by reducing tumor suppressor protein phosphatase and tensin homolog deleted on chromosome 10 (PTEN) [40]. Overexpression of mmu-miR-21-5p is seen to inhibit PTEN expression with an increase in the PI3/Akt pathway, leading to renal cell hypertrophy and fibronectin expression [40]. Overall it is clear that the effect of the miRNAs on these functions and pathologies is significant and important. Many of these miRNAs have been shown to be associated with features of the diabetes phenotype (insulin secretion or sensitivity) and the development of diabetic kidney disease. Many of these miRNAs have the potential to serve as biomarkers in clinical, human disease, an area that was extensively reviewed recently [41,42]. We summarize some of the studies that investigate the role played by miRNAs in the pathogenesis of diabetic nephropathy in Table 4. 

### 7.2. Hypertension

Hypertension is a major risk factor for developing coronary artery disease, congestive heart failure, sudden death [43], left ventricular hypertrophy [44], and stroke [45]. Coronary artery disease and stroke are the two major causes of death in the U.S. [46]. Hypertension is more prevalent in patients with chronic kidney disease (CKD) and is thought to be the second most common cause of end-stage renal disease in the U.S. [47]. Genetic, environmental, hemodynamic, renal, and hormonal factors have been implicated in the pathogenesis of hypertension. miRNAs are involved in nearly all pathophysiological alterations that underlie the development of hypertension and its cardiovascular and renal complications.

Oxidative stress due to inhibition of nitric oxide (NO) production and generation of reactive oxygen species could be the final common pathway for hypertension development [48]. Production of reactive oxygen species (ROS) may be influenced by specific miRNAs. In experimental models of oxidative stress (ROS generation, hydrogen peroxide exposure), apoptosis of human umbilical vein endothelial cells (HUVECs) was observed in a dose-dependent manner with concomitant increase in miR-210 levels [49]. Overexpression of hsa-miR-210-5p resulted in inhibition of apoptosis and decreased the concentration of reactive oxygen species. Thus, hsa-miR-210-5p may prevent the deleterious effects of ROS [49]. hsa-miR-155-5p was shown to directly inhibit endothelial nitric oxide synthase (eNOS) production by binding to the 3′ UTR of its mRNA, leading to increased oxidative stress [50]. Furthermore, simvastatin decreased hsa-miR-155-5p expression, thus restoring endothelium-dependent vasorelaxation, an effect that was independent of cholesterol levels [50]. Inhibition of miR-155 may be a therapeutic target for improving endothelial dysfunction and may even underlie some of the non-cholesterol (pleiotropic) effects of statins [50].

miRNAs may also play a role in the development of hypertension, by their effects on vascular smooth muscle cells (VSMCs). Aberrant division of VSMCs leads to vascular luminal hypertrophy and luminal narrowing which causes and propagates hypertension. hsa-miR-143-5p and hsa-miR-145-5p ensure proper development and regulation of VSMCs [51]. VSMCs deficient in hsa-miR-143-5p and hsa-miR-145-5p did not respond to vasocontractile stimuli but had increased synthetic activity [51]. These miRNAs played a critical role in the class switching of VSCMs from a synthetic unit to a vasocontractile unit [51].

Activation of renin-angiotensin-aldosterone systems (RAS) plays a cardinal role in pathophysiology and maintenance of different forms of hypertension. Activation of the angiotensin 1 receptor (AT1R) by Angiotensin II (Ang II) increases blood pressure by vascular smooth muscle cell proliferation, vascular constriction, cardiac remodeling, aldosterone production, and sodium retention, which plays a central role in the pathogenesis of hypertension [52]. These angiotensin mediated processes are under miRNA control. mmu-miR-155-5p inhibits AT1R expression and VSMC proliferation [53]. hsa-miR-29b-5p, hsa-miR-129-3p, hsa-miR-132-5p and hsa-miR-212-5p were upregulated by Ang II in human cell culture (HEK293N) [54]. miR-483-3p expression downregulated angiotensinogen and angiotensin-converting enzyme (ACE) and could be a novel therapeutic agent for hypertension management [55]. Inhibitors of the angiotensin-converting enzyme inhibitors (ACEi) have been shown to decrease renal disease progression in early diabetic nephropathy in type 1 and type 2 diabetes mellitus [43] and in preventing coronary artery disease and strokes [56]. Angiotensin-converting enzyme inhibitors have become a mainstay for the therapy of hypertension [57]. Some of the beneficial effects of ACEi could be mediated by suppression of miR-324-3p. In the Munich Wistar Fromter (MWF) rat model, which develops spontaneous progressive nephropathy, ACEi suppresses rno-miR-324-3p and attenuates the development of hypertensive nephropathy [58].

Sympathetic nervous system overactivity is one of the mechanisms for development and maintenance of hypertension. The role of the sympathetic nervous system (SNS) and RAS in the maintenance of hypertension was studied in mice which were genetically prone to develop hypertension (BPH/2J mice) [59]. Ganglion blocker use (SNS suppressor) in mice that were pre-treated with an ACEi (RAS suppressor) showed that hypertension in the BPH/2J was primarily mediated by the sympathetic nervous system during the active periods and RAS system during the inactive periods [60]. During active periods, BPH/2J mice had higher renal *Ren1* (Renin) mRNA and lower miR-181a indicating SNS mediated release of renin [60]. These findings suggest that mmu-miR-181a-5p inversely regulates the *Ren1* mRNA. The authors postulated that mmu-miR-181a-5p suppression potentiates sympathetic nervous system-mediated increase in renin production in BPH/2J mice during the active periods [60]. These findings were confirmed by a human study of mRNA and miRNA expression profiles in renal biopsies of hypertensive patients that showed hsa-miRNA-181a inversely regulated the *Ren1* mRNA [61].

Various animal models have been developed to study the effects of hypertension on kidneys. Dahl salt-sensitive (Dahl-SS) rats develop hypertension with medullary interstitial fibrosis when exposed to a high salt diet. Consomic SS-13^BN^ rats are genetically modified Dahl-SS rats that have less pronounced blood pressure rise and medullary interstitial fibrosis when exposed to a high salt diet [62]. Liu et al. studied miRNA expression profiles in these two rat models and showed that a high salt diet resulted in upregulation of miR-29b in Consomic SS 13^BN^ rats but not in Dahl-SS rats [33]. Various collagen genes that promote fibrosis were upregulated in Dahl-SS rats but not in Consomic SS 13^BN^ rats—a pattern opposite of miR-29b expression. Furthermore, a miR-29b knockdown Consomic SS 13^BN^ rat model had upregulation of various collagen genes, suggesting that miR-29b expression protects rats from hypertension-associated renal injury [33].

Kidney biopsies in patients with hypertension reveal glomerulosclerosis, tubular atrophy, interstitial fibrosis, and vascular smooth muscle cell hypertrophy. In a recent study, intrarenal miRNA expression profiles of 30 patients with hypertensive nephrosclerosis were compared to 20 normal controls. hsa-miR-200a-5p, hsa-miR-200b-5p, hsa-miR-141-5p, hsa-miR-429-5p, hsa-miR-205-5p, and hsa-miR-192-5p were significantly increased in patients with hypertensive nephrosclerosis [63]. Table 5 summarizes the studies exploring the role of miRNAs in the pathogenesis of hypertension.

### 7.3. Glomerulonephritis

Glomerulonephritides (inflammation of glomeruli) are a group of diverse disorders that may present as proteinuria and or/hematuria with renal dysfunction. Kidney biopsy findings include podocyte injury, mesangial and endocapillary proliferation, and disruption of basement membranes leading to focal and segmental glomerulosclerosis, tubular atrophy, and interstitial fibrosis. We will now discuss the role of miRNAs in some of the conditions that can cause glomerulonephritis.

#### 7.3.1. Focal Segmental Glomerulosclerosis

Focal segmental glomerulosclerosis (FSGS) is a pattern seen on kidney biopsy characterized by involvement of some of the glomeruli, with part of the involved glomerulus showing obliteration of the capillary lumen and increase in mesangial matrix. FSGS is usually caused by infections, medications, and conditions that cause chronic renal injury. At times, no cause of FSGS is found, and this is labeled as primary FSGS. A molecule that increases the permeability of glomerular basement membranes has been postulated in the pathogenesis of primary FSGS [64,65] but the exact nature of that molecule remains elusive. Podocyte injury is considered an inciting event in the development of FSGS.

In a puromycin-induced FSGS rat model, researchers found diminished rno-miR-30s-5p [66]. Replacement of rno-miR-30s-5p resulted in resolution of podocyte injury and proteinuria [66]. Furthermore, a human cell culture with hsa-miR-30a overexpression had amelioration of the cytoskeletal damage and apoptosis induced by puromycin or TGF-β treatment [66]. Glucocorticoid use caused sustained expression of rno-miR-30s-5p in podocytes [66]. Therefore, miR-30a plays a role in podocyte health and maintenance of cytoskeletal integrity. The histological findings in this model recapitulated the abnormal morphology in the mmu-miR-30a deficient Drosha and Dicer knockout podocyte models that were discussed previously [11,12,13].

Recently, the role of Brain Derived Neurotrophic Factor (BDNF) in maintaining the integrity of podocyte layer was studied [67]. Mice podocyte cell culture exposed to BDNF showed increased actin polymerization and increased number and length of podocyte foot processes with an associated increase in mmu-miR-132-5p and decrease in mmu-miR-134-5p. Overexpression of mmu-miR-132-5p and silencing of mmu-miR-134-5p in a podocyte cell culture also showed podocyte cell growth [67]. Furthermore, BDNF repaired podocyte damage in in vivo and in vitro models [67]. This study showed that BDNF maintains and repairs podocyte layer and this effect is mediated by increased expression of mmu-miR-132-5p and decreased expression of mmu-miR-134-5p. This agent may be used as a therapeutic agent in conditions associated with podocyte damage such as FSGS, minimal change disease and diabetic nephropathy.

miRNAs have also been used as biomarkers—both in serum and urine—to assess FSGS disease activity. In one study, researchers found elevated plasma hsa-miR-125b-5p, hsa-miR-186-50 and hsa-miR-193a-3p in patients with FSGS with area under curve (AUC) of 0.88, 0.78, and 0.91, respectively [68]. Patients in remission had lower hsa-miR-125b-5p and hsa-miR-186-5p concentrations [68]. These miRNA levels remained unchanged in patients that did not achieve remission. hsa-miR-186-5p levels also correlated with proteinuria [68]. Patients with FSGS and minimal change disease had higher urinary hsa-miR-200c-5p levels [69]. hsa-miR-196a-5p, hsa-miR-30a-5p and hsa-miR-490-5p were associated with FSGS disease activity [70]. Urinary hsa-miR-30a-5p was a weak predictor of steroid responsiveness in patients with active FSGS [70]. Table 6 summarizes some of the studies looking at the role of miRNAs in FSGS.

#### 7.3.2. IgA Nephropathy

IgA nephropathy is the most prevalent primary glomerulonephritis in the world. Abnormal O-galactosylation of IgA causes the formation of IgA complexes against which IgG and IgA are formed with deposition in the kidneys and activation of the complement system leading to kidney injury.

Genome-wide analysis has revealed various miRNAs which might play a role in IgA nephropathy. miRNA expression of 6 patients, each with biopsy-proven IgA nephropathy, compared to those with renal cell carcinoma, revealed upregulation of 11 miRNAs and downregulation of 74 miRNAs in the IgA nephropathy group [71]. Members of the hsa-miR-200 and hsa-miR-29 families, which regulate EMT and development of tissue fibrosis, showed prominent expression changes in patients with IgA nephropathy and were associated with tissue fibrosis and proteinuria [71].

miRNA hsa-let-7a-5p and hsa-miR-148b-5p control *N*-acetylgalactosaminyltransferase 2 (GALNT2) and 1 β 1,3 galactosyltransferase 1 (C1GALT1), respectively—these enzymes play a central role in aberrant IgA galactosylation. It has been shown that these enzymes are overexpressed in the peripheral blood mononuclear cells of IgA nephropathy patients [72,73].

miRNA expression profiles in paraffin embedded kidney biopsy specimens and urine from patients with IgA nephropathy was studied [74]. Both the renal tissue and urine of patients with IgA nephropathy exhibited increased expression of hsa-miR-146a-5p and hsa-miR-155-5p than controls. hsa-miR-146a-5p and hsa-miR-155-5p were inversely related with GFR and positively related with proteinuria. hsa-miR-155-5p expression was also associated with renal fibrosis. The possible mechanistic roles of these miRNAs in the pathogenesis of IgA nephropathy was further studied. Urinary hsa-miR-155-5p was inversely associated with interleukine 1 beta (IL-1β) and tumor necrosis factor alpha (TNF-α) and positively related with expression of Forkehad Box P3 (FOXP3—a transcription factor for regulatory T cell development) and regulated upon activation, normal T-cell expressed and secreted (RANTES—a chemokine). It was postulated that hsa-miR-146a-5p and hsa-miR-155-5p regulate the expression of pro-inflammatory molecules in patients with IgA nephropathy [74]. hsa-miR-155-5p has been shown to influence the regulatory T cell development [75,76]. Decreased regulatory T cell number/function has been implicated in the pathogenesis of IgA nephropathy [77]. Table 7 summarizes studies investigating the role of miRNAs in IgA nephropathy.

#### 7.3.3. Lupus Nephritis

Systemic lupus erythematosus (SLE) is a systemic disease due to dysregulated immune system activity. Kidney involvement in SLE often leads to chronic kidney disease and eventually kidney failure if left untreated, and it is the major cause of morbidity and mortality. Genetic factors have been implicated in SLE pathogenesis, but the underlying control mechanisms remain poorly defined.

Various lines of evidence point towards the role of miRNAs in SLE. miRNAs regulate 72 genes labeled as “autoimmune genes” that control various aspects of the immune system [78]. hsa-miR-181-5p, hsa-miR-186-5p, and miR-590-3p regulate more than 50% of the genes that are known to be differentially expressed in SLE patients [78]. Epstein-Barr virus (EBV) infection could be one of the initiating agents responsible for dysregulated immune response in SLE. EBV affects SLE patients more commonly with an increased number of infected peripheral white cells than healthy controls [79]. The exact causal link between EBV infection and SLE is not known; however, molecular mimicry is suspected. EBV latent membrane protein 1 activates hsa-miR-155-5p transcription through the nuclear factor κβ (NF-κβ) pathway [80]. miR-155 is expressed in regulatory T cells [81] and macrophages and promotes the development of inflammatory T cells [82]. B6.MRLc1 mice exhibit an immune complex-mediated glomerulonephritis with proliferative lesions that progress to glomerulosclerosis, tubular atrophy, and interstitial fibrosis [83]. These lesions showed expression of mmu-miR-146a-5p which increased with age, suggesting that it plays a role in renal inflammation [83]. Kidney biopsy analysis of patients with lupus nephritis showed upregulation of hsa-miR-146a-5p and hsa-miR-198-5p in the glomerular lesions and hsa-miR-638-5p in tubulointerstitial lesions [84]. In this study, the degree of interstitial miR-638 expression was significantly correlated with clinical markers of kidney damage (proteinuria) and the disease activity score [84]. Conversely, glomerular hsa-miR-146a-5p correlated with clinical markers of renal function (estimated glomerular filtration rate) and the disease activity score [84]. Hence, these two miRNAs may play a pathogenic role in the development of clinical lupus nephritis.

The role of miRNAs in the pathogenesis of proliferative lupus nephritis has been a subject of intense research. A high-throughput analysis of paraffin embedded kidney biopsies of pediatric patients showed differential expression of various miRNAs [85]. hsa-miR-26a-5p, hsa-miR-30b-5p, and hsa-miR-4286-5p, which are involved in cell cycle regulation, were decreased in the kidney biopsy samples and were studied further in a human mesangial cell model. Silencing of hsa-miR-26a-5p, hsa-miR-30b-5p, and hsa-miR-4286-5p resulted in increased expression of genes involved in cell cycle regulation. It has been shown previously that Trastuzumab, a monoclonal antibody against the human epithelial growth factor receptor 2 (HER-2), causes the arrest of the cancer cells in G1 phase with up-regulation of hsa-miR-26a-5p and hsa-miR-30b-5p [86]. The authors studied the effects of Trastuzumab on the human mesangial cell model and found that the mesangial cells treated with this agent have increased hsa-miR-26a-5p and hsa-miR-30b-5p expression. This lead to the hypothesis that HER-2 might be responsible for increased cell proliferation in lupus nephritis. This was validated in paraffin fixed human kidney biopsy specimens from patients with lupus nephritis that showed increased HER-2 expression in tubular, glomerular and mesangial compartments [85]. These findings were replicated in the glomeruli of a mouse lupus model. The factors that lead to increased expression of HER-2 in lupus nephritis were further studied. Human mesangial cells exposed to IFNα had increased expression of HER-2 than control cells. The authors found that urinary HER-2 levels are elevated in patients with lupus nephritis than in healthy controls. HER-2 levels were inversely related to lupus activity and correlated with proteinuria. Therefore, it was shown that INFα increases the HER-2 expression that leads to decreased hsa-miR-26a-5p and hsa-miR-30b-5p expression resulting in activation of the cell cycle leading to proliferative lupus nephritis. Table 8 summarizes the studies that investigate the role of miRNAs in the development of lupus nephritis.

#### 7.3.4. Anti-Neutrophilic Cytoplasmic Antibodies Associated Vasculitis (ANCA)

ANCA vasculitis is a small vessel vasculitis involving the kidneys as well as other organs and is characterized by the presence of either anti-Proteinase 3 (PR3) or anti-Myeloperoxidase (MPO) (components of neutrophils) antibodies. Currently, it is not known whether these antibodies are pathogenic and what are the inciting factors for production of these antibodies.

Pooled plasma samples from 40 patients who had active ANCA vasculitis or were in remission showed up-regulation of hsa-let-7f-5p and hsa-miR-424-5p, and downregulation of hsa-miR-106b, hsa-miR-9-5p, hsa-miR-125a-50, and hsa-miR-15b-5p [87]. These miRNAs regulate various aspects of the immune system [87], suggesting a direct role in the development of clinical disease.

hsa-miR-155-5p is upregulated in patients with ANCA-associated crescentic GN [88]. Nephrotoxic nephritis is a mouse model of ANCA vasculitis developed by injecting rats with rabbit or duck nephrotoxic sera [89] and has been found to closely correlate with human renal ANCA vasculitis [90]. A mmu-miR-155-5p knockout in this mouse model exhibited less severe lesions [88]. It was noted that miR-155 mediates the TH17 immune response and thus may be a therapeutic option for ANCA associated crescentic GN [88]. Table 9 summarizes the studies that investigate the role of miRNAs in ANCA vasculitis.

#### 7.3.5. Systemic Sclerosis (Scleroderma)

Systemic sclerosis is a condition associated with multiple organ fibrosis. It affects the kidney by causing thickening of the blood vessels, leading to hypertension, endothelial injury, and thrombotic microangiopathy. It has been shown that TGF-β [91] and miR-21 [92] are upregulated in systemic sclerosis. Furthermore, TGF-β regulates the expression of hsa-miR-21-5p and fibrosis-related genes, and hsa-miR-21-5p is inversely associated with Smad7 expression and may, therefore, be a therapeutic target for this condition [93]. Table 10 summarizes the results of a study investigating the role of miR-21-5p in scleroderma.

#### 7.3.6. Autosomal Dominant Polycystic Kidney Disease (ADPKD)

ADPKD is a disease characterized by impaired ciliary function leading to kidney and liver cyst formation and kidney failure in the vast majority of patients.

In the Sprague-Dawley rat model of ADPKD, 29 miRNAs were downregulated and only 1 miRNA (miR-21) was upregulated. Most of the dysregulated miRNAs control cell-to-cell interaction and crosstalk [94]. Global gene-expression studies in embryonic kidneys in an animal PKD model found differential expression of rno-miRs-10a-5p, rno-miR-30a-5p, rno-miR-96-5p, rno-miR-126-5p, rno-miR-182-5p, rno-miR-200a-5p, rno-miR-204-5p, rno-miR-429-5p and rno-miR-488-5p [95]. The mmu-miR-21-5p expression has also been associated with cyst progression [96]. Inhibition of mmu-miR-21-5p slows cyst growth in a mouse model of ADPKD [96]. The miR-17 cluster of miRNAs is upregulated in mouse models of PKD, and deletion of miR-17 cluster results in resolution of cysts, and better renal and animal survival [96]. We summarize the results of a study investigating the miRNA expression profile in ADPKD in Table 11.

#### 7.3.7. Alport Syndrome

Alport syndrome is due to abnormalities in genes encoding α3, α4 or α5 chains of collagen Type IV, resulting in abnormal basement membranes in the kidney, eyes, and inner ear. These changes in the kidney lead to abnormalities of glomerular basement membrane and progressive renal disease. mmu-miR-21-5p is preferentially expressed in the tubulointerstitium instead of glomeruli in normal mice; however, in the Col4 α3^−/−^ mice, mmu-miR-21-5p is expressed equally in both compartments [97]. As described previously, miR-21 has been associated with renal fibrosis. Introduction of anti-miR-21 oligonucleotides in Col4 α3^−/−^ mice resulted in the preservation of renal function, reduction in albuminuria, improved survival, reduced glomerulosclerosis, crescent formation, and tubular injury [97]. Currently, a Phase 1 clinical trial is being conducted to assess the safety, pharmacodynamics, and pharmacokinetics of a molecule that inhibits miR-21 (Table 12) [98].

## 8. miRNA Detection

**Figure 2:**
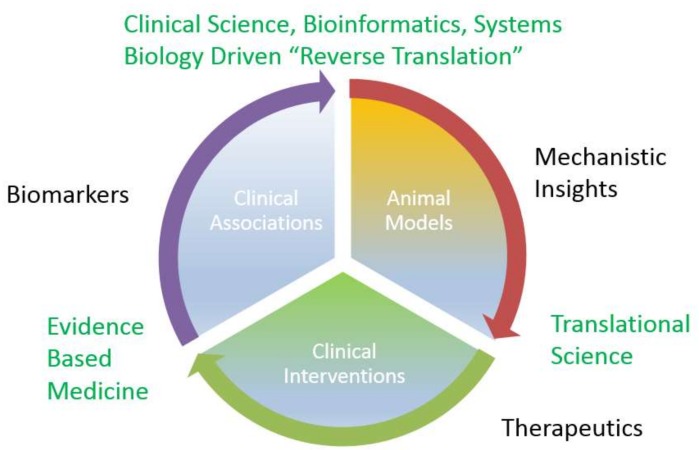
miRNA discovery cycle from biomarkers to therapeutics.

The preceding section highlights the role of specific miRNAs in normal renal development and physiology, but also the initiation and the progression of the interstitial fibrosis that underlies progressive forms of chronic kidney disease. It follows, that miRNAs detected in either plasma or urine, the two fluidic compartments directly affected by renal processing, may be mechanistically plausible, rational biomarkers for diverse forms of kidney diseases. In fact, miRNA associations found in observational human studies may offer a unique opportunity to “reverse translate” such findings into animal studies, which provide mechanistic insights into novel therapeutics that are tested in rigorous interventional clinical trials in humans. (Figure 2).

Nevertheless, detection of miRNAs poses unique challenges because of their short size and the similarity of many sequences to one another. These biochemical features of miRNAs may directly impact the performance of the three methods most commonly used for miRNA detection: quantitative real-time PCR (qPCR), microarrays, and next-generation sequencing (NGS). Each of these approaches comes with its distinct advantages, but also limitations when used as the basis for the development of miRNA biomarker assays.

Of the methods listed above, qPCR has the highest sensitivity, with a theoretical limit of detection of just a few copies per sample [99]. Several commercial kits are available for detection of miRNAs by qPCR, and although the specifics of each kit differ, they generally involve the addition of a known sequence to the 3′ end of the miRNA, followed by reverse transcription and PCR amplification using a miRNA-specific primer. Because each target of interest requires a separate PCR reaction and cannot easily be highly multiplexed, qPCR is less well-suited to high-throughput profiling than either microarrays or NGS. However, for targeted detection of a specific, small set of miRNAs, the cost of qPCR is comparatively low, and the hands-on time required is much less than that of other methods. Nevertheless, the high ionic strength of urine and the presence of urea (a non-specific inhibitor of the polymerase reaction [100]) may pose unique challenges when developing a urine-specific qPCR assay. Furthermore, the development of droplet digital PCR (ddPCR) more recently has improved the precision and reproducibility of qPCR measurements, especially for samples with low target abundance or high contaminant concentrations, and made absolute quantitation more accessible [101].

Microarray-based methods allow for the simultaneous measurement of many miRNAs, making them a better choice than qPCR for profiling a large set of targets. Commercial products are available covering all the mature miRNA sequences in miRBase on a single array. However, the amount of starting material required for microarray analysis is relatively high (~100ng per sample) and it remains difficult to design probes and hybridization conditions that can distinguish between closely related miRNA sequences. In addition, the dynamic range of microarrays is typically lower than either qPCR or NGS.

Unlike qPCR and microarrays, NGS requires no prior knowledge of the target sequences in the sample, and so is ideal for discovery studies. In addition, NGS is not affected by the complications of designing primers or probes with the specificity needed to distinguish between short sequences with high sequence identity. Consequently, as costs for NGS library preparation and sequencing have dropped, small RNA sequencing (sRNA-seq) has become widely used for miRNA profiling because of its ability to comprehensively interrogate the miRNAs (and other types of small non-coding RNAs) in a sample. As opposed to qPCR and microarrays, sRNA-seq allows the analysis of miRNAs with single nucleotide resolution, so not only can the canonical sequences be studied, but also variants arising from RNA editing or imprecise miRNA processing (isomiRs). sRNA-seq is not without drawbacks, however. Compared to qPCR and even microarray analysis, NGS is more expensive and more time intensive, both in sample preparation and data analysis. Most methods for preparing sRNA-seq libraries involve sequential ligation of adapters to the 3′ and 5′ ends of the miRNAs, followed by reverse transcription and PCR amplification to add indexes and other sequences needed for attachment to the flow cell and sequencing. The adapter ligation steps have previously been shown to introduce bias into small RNA libraries, which leads to decreased library diversity and prevents making quantitative comparisons of the expression level of different miRNAs in a sample [102,103,104]. Moreover, because the biases vary depending on the library preparation protocol, comparing data generated by two different sample prep methods is difficult [105,106]. Although attempts have been made to alleviate bias in sRNA-seq through modifications in library preparation methods [104,107,108] or to compensate for it during data analysis [109], it remains a significant issue that will need to be addressed in the future.

Several important issues affect all methods commonly used for measuring miRNAs. For example, whereas several housekeeping genes have been widely used for normalization in mRNA expression profiling studies, there is less consensus on similar invariant transcripts suitable for miRNA expression analysis. Other options include normalization based on sample input (input mass when practical or input volume when the amount of RNA in the sample is too low to reliably measure) or based on the addition of spike-in oligonucleotides added during sample processing. Normalization based on relative read counts (e.g., reads per million total miRNA reads or reads per million genome-mapped reads) is also frequently used to report sRNA-seq data, but this method can yield inaccurate results if samples have different proportions of small RNA species, either because of biological differences or technical differences such as variations in the size selection step of sRNA-seq library preparation.

Another issue affecting miRNA measurement is the lack of correspondence between data generated by different methods. It is not an uncommon practice to identify changes in miRNA expression using high-throughput methods such as microarrays or NGS and subsequently validate those changes by qPCR. However, it has been known for some time that changes in miRNA expression detected by one method are often difficult to corroborate across methods [110]. High among the problems here is that different methods exhibit different sequence-specific biases. Even within a given method, however, results may not be comparable if the data is generated using kits from different vendors, as mentioned above for sRNA-seq, and as previously shown for microarray and qPCR [111,112]. For all these reasons, measurement of miRNAs requires careful planning, care in precisely executing protocols, and repeated measurements when possible.

Measurement of extracellular miRNAs is further complicated by low concentration and inhibition by other macromolecules in the sample. Although there is great interest in profiling RNAs present in biofluids such as blood and urine to identify disease biomarkers, obtaining accurate and reproducible results from these sources remains challenging. The primary reason that measuring miRNA in biofluids is difficult is that the RNA concentration is much lower than in cells or tissues. Typical RNA isolation methods recover only about 10–50 ng of total RNA per milliliter of cell-free plasma, with even lower yields from fluids such as urine and saliva. In addition, the presence of inhibitors derived either from the sample itself or during the collection procedure and co-purified with the RNA, can be problematic for the enzymatic steps in library preparation. Salts present in urine samples and heparin used as an anticoagulant during plasma collection are two examples of inhibitors commonly encountered with biofluid samples. Furthermore, the handling and storage methods of the samples before RNA extraction can have a significant impact on the results. Lysis of cells during collection (hemolysis in plasma, for example) or incomplete removal of intact cells before RNA isolation can also significantly distort the miRNA profile from biofluids since cellular RNA content is much higher than that of the cell-free fluid. Thus, sample collection, RNA isolation, and library protocols are all critical for accurate profiling.

### miRNA as Personalized Diagnostics

**Figure 3:**
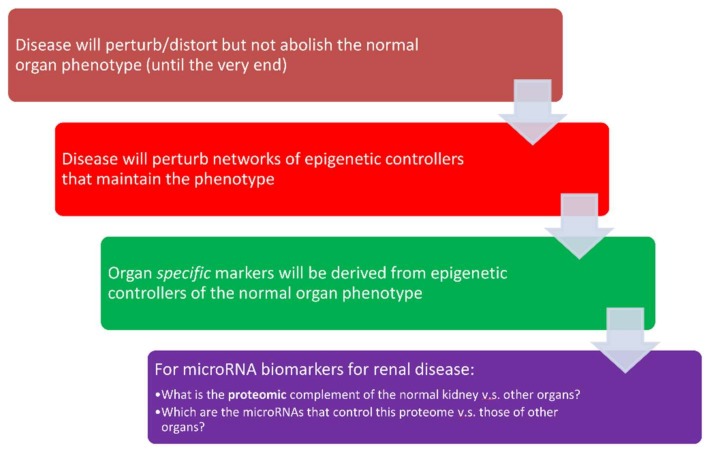
miRNAs as personalized diagnostics in kidney diseases.

miRNAs have a unique role among biological regulatory molecules, participating in feed-forward regulatory loops [113,114], whose connectivity (“fan-in” and “fan-out” patterns) differ from other control systems (e.g., transcription factors or kinases). Standard control theory of artificial systems informs us that such layouts are signatures of master controllers that fine-tune the performance of the system being controlled. Translated in biological terms, one should expect miRNAs to be tightly involved when exquisite sensitive control of a critical biological system and the corresponding phenotype(s) is required. In fact, the involvement of miRNAs in sodium and potassium regulation, provide examples of such a role, which is highly context-specific and sensitive to environmental stimuli. A corollary of these lessons from control theory is that miRNAs will likely prove to be successful candidates for the development of personalized diagnostics across the spectrum of kidney diseases (Figure 3). According to this hypothesis, disease processes will perturb the normal organ, but will nonetheless not abolish normal phenotype until very late in the disease process. Such a phenotypic resilience will be due to changes in the normal expression pattern of miRNAs that may be detected in biofluids accessible to the organ in question. The critical challenge for the development of personalized diagnostics is to develop organ-specific signatures whose alterations give clues to the presence or lack thereof of progressive organ dysfunction. Our previous research [115] in the area of diabetic kidney disease, illustrates the potential of miRNAs in the area of kidney disease. In particular, we discovered a signature of miRNAs that can predict the development of overt kidney damage, two years before it is detected by the conventional laboratory tests of serum creatinine and urinary albumin. The miRNAs in this signature were involved in the regulation of normal physiological processes and tissue fibrosis. Furthermore, the signature appeared to be relatively specific for diabetic kidney disease since it predicted less strongly the development of kidney damage in an independent cohort of patients.

## 9. Micro RNAs as a Therapeutic Option in Renal Diseases

In the previous sections, we discussed the roles of miRNAs in normal kidney function as well as kidney diseases associated with miRNA dysregulation. There is significant interest in the use of miRNAs as therapeutic agents since they modulate the activity of numerous genes. miRNA-based therapeutics are based on either inhibiting a deleterious miRNA or replacing a deficient beneficial miRNA. miRNA antagonists—also called antagomirs or antimiRs—are single-stranded molecules that are designed to bind directly to a mature miRNA and to block its action. Deficient miRNAs can be replaced by either making small interfering miRNAs (siRNAs), which are small double-stranded RNA molecules encapsulated in nanoparticles and delivered to the target site [116] or by viral vectors that express the desired miRNA.

### 9.1. miRNA Delivery

Designing an effective miRNA mimic and delivering it to its intended target organ without degradation or causing unintended effects has been the subject of intense research. Designing miRNA mimics that effectively block their targets without affecting any unintended transcripts has proven problematic [117]. Therefore, these molecules must be thoroughly tested to fully understand all their intended and unintended effects.

The process of delivering a miRNA molecule to its intended target is fraught with difficulties. Naked miRNAs are unstable in the blood and are rapidly degraded by the mononuclear macrophage system and removed from circulation by the kidneys and liver. Therefore, effective delivery methods that prevent miRNA degradation must be devised. These delivery vehicles should be non-toxic, have low immunogenicity, and should be able to deliver a large proportion of the miRNAs to their intended target [118]. Some of the techniques used to deliver miRNAs are shown in Table 13.

### 9.2. MicroRNA-Based Renal Therapeutics

Various phase I and phase II trials are underway or have been completed for miRNA-based therapeutic agents for the management of chronic hepatitis C, diabetes mellitus type 2 with fatty liver and cancers [116]. miRNAs are also being used as therapeutic agents in renal diseases. Table 14 shows a summary of miRNA-based therapeutics for the conditions affecting the kidneys.

Regulus Therapeutics in collaboration with Genzyme has developed a single-stranded molecule RG-012 that inhibits miR-21. In a rat model of Alport syndrome, miR-21 inhibition by this molecule led to milder kidney disease and improved survival than control mice. There was less glomerulosclerosis and tubulointerstitial fibrosis in the treated mice with no adverse events [97]. A phase I randomized, double-blinded, placebo-controlled study is currently being conducted to study the safety and efficacy of RG-012 in male subjects with Alport syndrome [98]. miRagen Therapeutics is developing a molecule MRG-201 that promotes miR-29 activity and thus modulates fibrosis. This molecule has potential roles in preventing progression of CKD in diabetic nephropathy, IgA nephropathy, and scleroderma. A phase 1 study to evaluate the safety and tolerability of this agent has been completed [130].

## 10. Conclusions

It has been shown in practice over the past decade that extracellular miRNAs can provide informative biomarkers for multiple biological effects and pathologies. The value of understanding miRNA function, however, is much broader. In concert with the multiple factors regulating transcription, miRNAs provide an additional level of control of gene expression, largely at the post-transcriptional level. Their influence on various biological pathways is both widespread and complex and is often subtle. In the last 20 years, tremendous progress has been made in understanding their roles in renal physiology and pathology, and this is beginning to open several new lines of investigation. Research is currently underway to study and modulate miRNAs specifically to control maladaptive repair that leads to fibrosis in various renal diseases. miRNAs also provide us a novel opportunity to develop new ways of studying disease activity and to assess the efficacy of therapeutic agents. Since miRNAs can be targeted directly, although this is sometimes difficult in practice, they provide the opportunity to develop a new class of therapeutic agents. miRNA-based diagnostics and therapeutics, therefore, have the potential to lead medicine into a new era of effectiveness.

## Figures and Tables

**Table 1:** Summary of the studies investigating the role of miRNAs in renal development.

Source/Reference	Study Sample/miRNA Detection Method	Target/Aim	Results
Mouse [6]	Embryonic, Renal tissue,	Conditional Dicer deletion in the nephron progenitor cells	↓ number and ↑ apoptosis of the nephron progenitor cells
	Microarray-based method		↑ expression of mmu-miR-10a, mmu-miR-17-5p, and mmu-miR-106b
Mouse [8]	Embryonic, Renal tissues on paraffin, Real Time-PCR (RT-PCR)	Conditional Dicer deletion in the ureteric bud	Disruption of ciliogenesis in the ureteric bud, Small and cystic kidneys with hydronephrosis, mmu-miR-30b expression in the nephron progenitor cells
Mouse [9]	Embryonic, Renal tissue on paraffin sections, RT-PCR	Deletion of the mmu-miR-17~92 cluster in the nephron progenitor cells	Pre-natal: Preservation of the nephron progenitor cells but impaired proliferation
			Post-natal: ↓ nephron number, Albuminuria by 6 weeks, Podocyte effacement and Focal Segmental Glomerulosclerosis by 3 months
Mouse [10]	Embryonic, Renal tissue, Microarray-based method	Conditional deletion of Dicer in the maturing renal tubules	Pre-natal: Down-regulation of mmu-miR-200b/c, Upregulation of Pkd1
			Post-natal: Tubular and glomerular cyst formation
Mouse [11]	Embryonic, Renal tissue on paraffin, RT-PCR	Conditional deletion of Dicer in the podocytes	Post-natal: Proteinuria by 2 weeks of birth, Glomerular and tubular injury by 4 weeks
			↓ expression of slit diaphragm proteins
			↑ glomerular expression of mmu-miR-23b, mmu-miR-24, and mmu-miR-26a
Mouse [13]	Embryonic and after birth, Renal tissue, RT-PCR	Conditional deletion of Drosha in 1. Pre-natal period, 2. Post-natal in 2-3 months old mice	Collapsing glomerulopathy was seen in the pre-as as well as post-natal inactivation of Drosha

↑ Increased; ↓ Decreased.

**Table 2:** Summary of studies investigating the role of miRNAs in renal physiology.

Source/Reference	Study Sample/miRNA Detection Method	Target/Aim	Results
Mouse [14]	Embryonic, Renal tissue on Paraffin, RT-qPCR	Conditional deletion of Dicer in the renal renin-producing cells	Severe depletion of Juxta-Glomerular cells (JG) cells, Absent mmu-miR-145a-5p in JG cells, ↓ Blood pressure (BP) in the dicer knockout mice, ↓ Kidney size, Renal vascular abnormalities, and strip fibrosis
Mouse [15]	Adult digested pooled renal tissue, RT qPCR	mmu-miR-192-5p suppression by antagomir injections	↑ Urine output in mice that were fed a high Na diet
			↑ Na^+^/K^+^ ATPase β-1 subunit
Mouse [16]	Adult, microdissected renal tissue, RT-PCR, and microarray method	Effects of aldosterone on the expression of mmu-miR-192-5p and WNK1	Stimuli that ↑ aldosterone was associated with ↓ mmu-miR-192-5p and ↑ WNK1 expression
Mouse [17]	Adult homogenized renal tissue, RT-PCR	Effects of high K diet on mmu-miR-802 in the cortical collecting duct	↑ mmu-miR-802, ↓ Caveolin-1 which suppresses ROMK
Mouse [18]	Adult, Renal tissue-immunomagnetic separation method to isolate the thick ascending limb cells from the mouse kidney, RT-PCR	Effects of mmu-miR-9-5p and mmu-miR-374 on claudin 14, gene expression in the thick ascending limb of the loop of Henle	mmu-miR-9-5p and mmu-miR-374 suppress claudin-14

↑ Increased; ↓ Decreased.

**Table 3:** Studies investigating the role of miRNAs in renal fibrosis and maladaptive repair.

Source/Reference	Study Sample/miRNA Detection Method	Target/Aim	Results
Human [25]	Proximal Tubule HK-2 cell culture, RT-PCR	Identify miRNAs that post-transcriptionally modify TGF-β1	hsa-miR-744-5p post-transcriptionally inhibits expression of the TGF-β1 ligand
Rat [26]	Proximal tubular epithelial cell culture, RT-PCR	rno-miR-200a-5p expression and its role as translation repressor of TGF-β signaling	rno-miR-200a-5p decreased TGF-β2 expression
Rat and Human [27]	Rat kidney tubular epithelial and mesangial cell lines, human immortalized podocytes, RT-PCR	To assess the expression of miRNAs in renal tissue exposed to TGF-β	↓ rno-miR-192-5p and rno-miR-215-5p expression
		To study the role of miRNAs in E-Cadherin expression	↓ E-Cadherin expression via suppression of Zinc finger E-box-binding homeobox 2 (ZEB2) mRNA by rno-miR-192-5p and rno-miR-215-5p
			↑ expression of extra cellular matrix proteins
Human [31]	Formalin-fixed renal tissue in patients with diabetic nephropathy, RT-PCR	Association of hsa-miR-192-5p expression in the kidney biopsy with the severity of diabetic nephropathy	hsa-miR-192-5p expression inversely related to tubulointerstitial fibrosis and low eGFR
Human and Mouse [32]	Skin biopsy samples of the patients with systemic sclerosis (SSc), bleomycin-induced skin fibrosis mouse model, RT-PCR	To investigate the role of miRNA as posttranscriptional regulators of profibrotic genes in systemic sclerosis	↓ hsa-miR-29a-5p in SSc patients was associated with ↑ profibrotic proteins
			↑ mmu-miR-29a-5p in bleomycin-induced fibrosis
Rat [33]	Adult, Renal medulla, microarray-based method, and RT-PCR	Expression of miRNAs in the renal medulla of rats that spontaneously develop hypertension when exposed to a high salt diet	Up-regulation of mmu-miR-29b-5p prevents hypertension associated medullary interstitial fibrosis
Human and Mouse [34]	Cultured human and mouse mesangial cells, microarray-based method, and qPCR	miRNA profile in the cells exposed to high glucose and TGF-β	↑ miRNA-377 relative to controls
			miRNA-377 ↑ fibronectin protein indirectly
Human [35]	Primary human fibroblasts culture and transplant recipient kidney biopsy samples (formalin-fixed and paraffin-embedded) that had chronic allograft nephropathy, Microarray-based method	To study the role of hypoxia in the miRNA expression profile	Hypoxia caused ↓ hsa-miRNA-449a-5p and ↑ SERPINE1 gene expression. SERPINE1 protein was demonstrated in areas of renal fibrosis in the kidney biopsy

↑ Increased; ↓ Decreased.

**Table 4:** Mechanistic, experimental studies investigating the role of miRNAs in diabetic nephropathy.

Source/Reference	Study Sample/miRNA Detection Method	Target/Aim	Results
Mouse [36]	Conditionally immortalized mouse podocytes, real-time PCR, and microarray-based method	miRNA expression profile in a diabetic mouse model as well as in mouse podocytes exposed to hyperglycemia.	mmu-miR-29c-5p elevated in mouse models relative to controls.
			↑ mmu-miR-29c-5p in podocytes exposed to hyperglycemia
		To identify the target of miRNA in these models	mmu-miR-29c-5p inhibits Spry1 gene and thus promotes apoptosis
			mmu-miR-29c-5p activates Rho kinase, which ↑ apoptosis and fibronectin deposition by inhibiting the Spry1
Human [31]	Human proximal convoluted tubule cell line culture, kidney biopsy samples of patients with established diabetic nephropathy, microarray-based method	To study the expression profile of miRNA in a PCT cell culture under high glucose conditions, miR expression profiling in pooled RNA from formalin-fixed, paraffin-embedded tissue from renal biopsies	TGF-β treatment ↓ hsa-miRNA-192-5p in vitro. ↓ hsa-miRNA-192-5p expression on kidney biopsy was associated with ↑ tubulo-interstitial fibrosis
Mouse [38]	Mouse kidney cell culture, Diabetic mouse, real-time PCR	To study the processes that result in TGF-β mediated development of diabetic nephropathy	TGF-β ↑ mmu-miR-216a-5p and collagen type I α1
Mouse [39]	Diabetic mouse model.	Interaction between TGF-β and miRNAs in the development of diabetic nephropathy	mmu-miR-192-5p and mmu-miR-200b/c-5p ↑ TGF-β1
			TGF-β1 exposure lead to ↑ mmu-miR-200b/c
			mmu-miR-192-5p expression lead to ↑ mmu-miR-200b/c-5p
	Primary mouse mesangial cell culture, real-time PCR		miR-192 ↑ TGF-β1 promotor activity and ↓ TGF-β1 repressor activity
			TGF-β1 ↑ Col1α2 and α4 activity causing ECM accumulation
Mouse [40]	Renal cortical tissue from a diabetic mouse model, rat mesangial cell culture, human mesangial cell culture, real-time PCR	miRNA profile of mouse cortical cells and rat and human cultured mesangial cells exposed to a high glucose environment	↑ mmu-miR-21-5p expression in mice cortical tissue
			↑ miR-21-5p inhibited PTEN expression with an increase in the PI3/Akt pathway, leading to renal cell hypertrophy and fibronectin expression in human and rat mesangial cell culture

↑ Increased; ↓ Decreased.

**Table 5:** Studies investigating the role of miRNAs in hypertension.

Source/Reference	Study Sample/miRNA Detection Method	Target/Aim	Results
Human [49]	Human umbilical vein endothelial cells (HUVECs), qRT-PCR	Role of hsa-miR-210-5p in HUVECs under oxidative stress	hsa-miR-210-5p prevented deleterious effect of the reactive oxygen species
Human [50]	HUVECs, Discarded human internal mammary arteries, qRT-PCR	Association of hsa-miR-155-5p with endothelial nitric oxide synthase(eNOS) activity	↑ hsa-miR-155-5p caused ↓ eNOS activity
			Simvastatin ↓ hsa-miR-155-5p and restored endothelium-dependent vasorelaxation
Mouse [51]	Various mouse tissues derived at various developmental stages, microarray-based method	Role of mmu-miR-143-5p and mmu-miR-145-5p in development of vascular smooth muscle cells	Vascular smooth muscle cells (VSCM) deficient in mmu-miR-143-5p and mmu-miR-145-5p did not respond to vasocontractile stimuli but had ↑ synthetic activity
Mouse [53]	Primary cultured VSMC from mice aorta, RT-PCR	Effect of mmu-miR-155-5p on Angiotensin II mediated VSMC proliferation	mmu-miR-155-5p antagonized the ANG II induced ↑ in VSMC viability
Human [54]	Cultured human cells (HEK293N)	Role of ANG II mediated miRNA regulation	↑ hsa-miR-29b-5p, hsa-miR-129-3p, hsa-miR-132-5p and hsa-miR-212-5p
Human, mouse, and rat [55]	Human, mice and rat cell cultures, microarray-based method	To study the Angiotensin 1 receptor (AT1R) regulated miRNA expression is VSMCs of various species	miR-483-3P expression ↓ angiotensinogen and angiotensin-converting enzyme (ACE)
Rat [58]	Microdissected glomeruli from Munich Wistar Frometer (MWF) rats, RT-PCR, and microarray-based method	miRNA expression profile	ACEi suppress rno-miR-324-3p and attenuates the development of hypertensive nephropathy
Mice [60]	Genetically hypertensive mice (BPH/2J), renal tissue	Role of renal angiotensin system and sympathetic nervous system in hypertension	mmu-miR-181a-5p suppression potentiates sympathetic nervous system-mediated increase in renin production in BPH/2J mice during the active periods
Human [61]	Renal biopsies of patients with hypertension who underwent nephrectomy for non-invasive renal cancer, microarray-based method, and qPCR	miRNA profile of patients with hypertensive nephrosclerosis	hsa-miR-181a inversely regulated the *Ren1* mRNA

↑ Increased; ↓ Decreased.

**Table 6:** Studies investigating the role of miRNAs in FSGS.

Source/Reference	Study Sample/miRNA Detection Method	Target/Aim	Results
Human and rat [66]	Kidney biopsy samples from patients with FSGS, Human podocyte culture, Rat renal tissue, RT-qPCR	To study the effect of TGF-B, and Puromycin on hsa-miR-30 and rno-miR-30	hsa-miR-30-5p expression ameliorated TGF-B mediated podocyte damage
			Puromycin caused decreased rno-miR-30a in rats, replacement of this miR resulted in resolution of proteinuria and podocyte injury in rats
Mouse, Zebra fish [67]	Mouse podocyte cell line, Zebra fish	To study the effects of brain derived neuropathic factor (BDNF) on podocyte miRNA expression profile and integrity	BDNF ↑ mmu-miR-132-5p and ↓ mmu-miR-134-5p and thus ↑ podocyte cell growth and repairs damage
Human [68]	Plasma samples of patients with proteinuria due to various etiologies, quantitative reverse transcription-polymerase chain reaction	Plasma miRNA profiles of patients with proteinuria due to FSGS (in relapse as well as in remission)	Patients with FSGS exhibit ↑ hsa-miR-125b-5p, hsa-miR-186-5p and hsa-miR-193a-3p
			↓ hsa-miR-125b-5p and hsa-miR-186-5p in patients in remission
Human [69]	Urine from patients with various glomerular diseases including FSGS	To assess urinary miRNA profile in patients with various types of glomerulonephritis (GN)	hsa-miR-200c-5p present in patients who had focal segmental glomerulosclerosis
Human [70]	Pooled urine from patients who had either active FSGS or were in complete remission, qRT-PCR	Urinary miRNA profile in patients with FSGS	↑ hsa-miR-196a-5p, hsa-miR-30a-5p and hsa-miR-490-5p in urine were associated with FSGS disease activity
			Urinary hsa-miR-30a-5p was a weak predictor of steroid responsiveness in patients with active FSGS

↑ Increased; ↓ Decreased.

**Table 7:** Studies investigating the role of miRNAs in IgA nephropathy.

Source/Reference	Study Sample/miRNA Detection Method	Target/Aim	Results
Human [71]	Genome-wide association study of miRNA expression profile in kidney biopsies of patients with IgA nephropathy compared to renal cell cancer, RT-qPCR	To identify miRNAs that may play a role in IgA nephropathy	In IgA nephropathy patients:
			↑ hsa-miR-133a-5p, hsa-miR-133b-5p, and hsa-miR-486-5p
			↓ hsa-miR-220 family, hsa-let-7a-5p, hsa-miR-628-5p, hsa-miR-195-5p, and hsa-miR-125b-5p
Human [72]	Peripheral blood mononuclear cells from patients with IgA nephropathy, RT-PCR	To study the association of hsa-miR-let-7b-5p with GLANT2 enzyme activity in patients with IgA nephropathy	↑ hsa-miR-let-7b-5p associated with ↓ GLANT2 levels
Human [73]	Peripheral blood mononuclear cells from patients with IgA nephropathy, Microarray-based method	To study the miRNAs that potentially target C1GALT1	↑ hsa-miR-148b-5p associated with ↓ C1GALT1 mRNA
			↑ hsa-miR-148b-5p associated with ↑ galactose-deficient IgA1
Human [74]	Kidney biopsy and urine specimens, RT-PCR	To study the renal and urinary miRNA expression profile in patients with IgA nephropathy	↑ hsa-miR-146a-5p and hsa-miR-155-5p
			These miRNAs were inversely related to GFR and positively related to proteinuria

↑ Increased; ↓ Decreased.

**Table 8:** Biopsy studies investigating the role of miRNAs in lupus nephritis.

Source/Reference	Study Sample/miRNA Detection Method	Target/Aim	Results
Mouse [83]	Paraffin-embedded kidney tissue, Microarray-based technique, and RT-PCR	To assess miRNA expression profile in a mouse model that spontaneously develops inflammation with age	↑ mmu-miR-146a-5p associated with increased kidney biopsy inflammatory score
Human [84]	Kidney biopsies of patients with lupus nephritis	To identify the miRNA expression profile in patients with lupus nephritis compared to healthy controls	In kidney biopsies of patients with lupus nephritis:
			↑ hsa-miR-146a-5p and hsa-miR-198-5p in the glomerular lesions
			↑ miR-638 in tubulointerstitial lesions
			↑ Interstitial hsa-miR-638-5p associated with proteinuria and disease activity score
			↑ Glomerular hsa-miR-146a-5p associated with eGFR and disease activity score
Human [85]	Paraffin-embedded kidney biopsy samples from pediatric patients, Human mesangial cell culture, Next generation sequencing, RT-qPCR	miRNA expression profile in the renal biopsy samples	↓ hsa-miR-26a-5p and hsa-miR-30b-5p in renal biopsy specimens of patients with lupus nephritis
		To study the role of hsa-miR-26a-5p and hsa-miR-30b-5p in human epidermal growth factor receptor 2 (HER2) regulation	Trastuzumab (HER-2 antagonist) exposure of the human mesangial cell line caused ↑ hsa-miR-26a-5p and hsa-miR-30b-5p
			IFNα → ↑ HER-2 expression in human mesangial cell line

↑ Increased; ↓ Decreased.

**Table 9:** Studies investigating the role of miRNAs in ANCA associated nephritis.

Source/Reference	Study Sample/miRNA Detection Method	Target/Aim	Results
Human [88]	Pooled plasma from patients with MPO and PR3 + ANCA vasculitis, RT-qPCR	To study the expression profile of miRNAs in patients with ANCA vasculitis	↑ hsa-let-7f-5p, hsa-miR-424-5p
			↓ hsa-miR-106b, hsa-miR-9-5p, hsa-miR-125a-50, and hsa-miR-15b-5p
Human and Mouse [89]	Renal tissue from patients with ANCA vasculitis, A mouse model of ANCA vasculitis, RT-qPCR	To study the role of miR-155 in T cell-mediated inflammation in a mouse model of ANCA vasculitis as well as humans with ANCA vasculitis	↑ hsa-miR-155-5p expression in crescents found in kidney biopsies of patients with ANCA vasculitis
			↑ mmu-miR-155-5p expression in crescents found in kidney biopsies of a mouse model of ANCA vasculitis
			Less severe lesions in mmu-miR-155-5p knockout mice

↑ Increased; ↓ Decreased.

**Table 10:** Study investigating the role of miRNAs in scleroderma-associated renal disease.

Source/Reference	Study Sample/miRNA Detection Method	Target/Aim	Results
Human [93]	Skin samples from patients with Scleroderma and normal controls, RT-qPCR	miRNA expression profile in patients with scleroderma compared to normal controls	↑ hsa-miR-21-5p in skin tissue as well as fibroblasts of patients with scleroderma

↑ Increased.

**Table 11:** Study investigating the role of miRNAs in autosomal dominant polycystic kidney disease.

Source/Reference	Study Sample/miRNA Detection Method	Target/Aim	Results
Rat [94]	Adult Rat model of PKD, kidney tissues, RT-qPCR	miRNA expression profiles in a rat model of PKD	Differential expression of rno-miRs-10a-5p, rno-miR-30a-5p, rno-miR-96-5p, rno-miR-126-5p, rno-miR-182-5p, rno-miR-200a-5p, rno-miR-204-5p, rno-miR-429-5p and rno-miR-488-5p

**Table 12:** Study investigating the role of miRNAs in Alport syndrome.

Source/Reference	Study Sample/miRNA Detection Method	Target/Aim	Results
Mouse [97]	Adult mouse model of Alport syndrome, kidney tissue, qPCR	To study the role of mmu-miR-21-5p inhibition in a mouse model of Alport syndrome	Anti-miR-21 oligonucleotides protect against kidney failure and increase survival in this mouse model

**Table 13:** miRNA delivery methods.

**Viral Vectors**	
Pathogenic genes are removed from the virus and are replaced by the miRNA gene. This modified virus makes a double-stranded miRNA mimic which associates with Ago proteins and forms the miRNA silencing complex. Adenovirus, adeno-associated virus, retrovirus, and lentivirus have been used as miRNA vectors. This approach is limited by low vector titers, high immunogenicity, the ability to work only in dividing cells, and clinical safety issues [119].	
**Nano-particles**	
Poly-Particles	Polylactic-co-glycolic acid (PLGA) particles are small polymers that have been used to deliver siRNAs, miRNAs and viral vectors [120]. They are non-toxic and have been used in clinical medicine for a long time. There is often poor loading of siRNAs and miRNAs although techniques are being developed to solve this problem [120].
Natural lipid emulsion	Natural lipid emulsions have been used to replace tumor suppressor genes in lung cancer. These particles are uncharged, do not make aggregates in the liver and are not scavenged by macrophages [121]. Questionable delivery of the siRNAs to the target site is an issue with this technique.
Cationic Lipid-based nano-liposomes	1,2-dioleoyl-*sn*-glycero-3-phosphocholine (DOPC) nano-liposomes have been found to be highly effective in delivering miRNAs [122].
Bacterial mini-cells	Bacterial mini-cells that are produced by inactivating genes involved in bacterial growth have been used to deliver chemotherapeutic agents [123]. A phase 1 study is currently ongoing to deliver miR-16 family miRNAs, which suppress tumor growth in malignant pleural mesothelioma and non-small cell lung cancer, using this technique [124].
Cationic polymers	Low molecular weight with a branched structure polyethyleneimine has been used for siRNA delivery [125].
Polyamidoamines	Initially designed for delivery of plasmids, polyamidoamines polymers have been used for siRNA delivery. These molecules can be designed precisely to the desired sizes and molecular weights [126].
Collagen-based molecules	Atelocollagen is a calf dermis derived type 1 collagen which has been used to deliver siRNA locally [127] as well as systematically [128].
Cyclodextrin polycation	siRNA, when complexed with cyclodextrin polycation delivery system, was shown to effectively silence the intended oncogene [129].

**Table 14:** Trials involving miRNAs in renal parenchymal disease.

Molecule	Therapeutic Agent/Mode of Action	Pharm* Company	Targeted Disease	Trial Description	Trial Results
RG-012	miR-21/Inhibits	Regulus/Genzyme	Alport Syndrome	Phase 1, Open-label, Multi-center study of the subjects with Alport syndrome, *n* = 10	Ongoing, Estimated completion date December 2018
MRG-201	miR-29/Promotes	Mirage	Scar tissue formation in skin, intended uses in Scleroderma, Diabetic nephropathy, and pulmonary fibrosis	Phase 1, Double-Blind, Placebo-Controlled, Single and Multiple Dose-Escalation Study to investigate the safety, tolerability, pharmacokinetics, and pharmacodynamics activity of MRG-201 following local intradermal injection in normal healthy volunteers, *n* = 54	Reduced fibrosis in humans who received MRG-201
RG-125/AZD4076	miR-103/107	AstraZeneca/Regulus	Type 2 diabetes and non-alcoholic steatohepatitis	Phase I/IIa to investigate the effect on whole-body insulin sensitivity, liver fat content, safety, and tolerability	Discontinued—June 2017

Pharm*: Pharmaceutical company.

## Abbreviations

3′ UTR	3′-Untranslated Region
ACE	Angiotensin-converting Enzyme
ACEi	Angiotensin-converting Enzyme Inhibitors
ADPKD	Autosomal Dominant Polycystic Kidney Disease
ANCA Vasculitis	Anti-Neutrophilic Cytoplasmic Antibodies associated Vasculitis
Ang II	Angiotensin 2
AT1R	Angiotensin 1 Receptor
BDNF	Brain Derived Neurotrophic Factor
BIM	Bcl-2-Like Protein 11
BP	Blood Pressure
C1GALT1	1-β-1,3-Galactosyltransferase-1
CCD	Cortical Collecting Duct
Col4 α3^−/−^	Homozygous for Collagen Type 4 alpha 3 Chain Absence
Consomic SS-13^BN^	Consomic Salt Sensitive Rats
Dahl-SS	Dahl Salt-Sensitive
DCT	Distal Convoluted Tubule
ddPCR	Droplet Digital Polymerase Chain Reaction
DOPC	1,2-Dioleoyl-*sn*-Glycero-3- Phosphocholine
ECM	Extra Cellular Matrix
eGFR	Estimated Glomerular Filtration Rate
EMT	Epithelial to Mesenchymal Transition
ENaC	Epithelial Sodium Channel
eNOS	Endothelial Nitric Oxide Synthase
ESRD	End-stage Renal Disease
FF	Feed Forward
FOXP3	Forkhead Box P3
FSGS	Focal Segmental Glomerulosclerosis
GALNT2	*N*-Acetylgalactosaminyltransferase 2
GN	Glomerulonephritis
HUVECs	Human Umbilical Vein Endothelial Cells
IL-1β	Interleukin 1 Beta
isomiRs	Imprecise miRNA Processing
miRNA	Micro RNA
MPO	Myeloperoxidase
mRNA	Messenger Ribonucleic Acid
MWF rat model	Munich Wistar Fromter (MWF) Rat Model
NCC	Sodium Chloride Co-Transporter
NF-Κβ	Nuclear Factor Kappa Beta
NGS	Next-Generation Sequencing
NO	Nitric Oxide
PI-3/Akt	Phosphatidylinositol-3 Kinase/Akt Pathway
PKD1	Polycystic Kidney Disease 1
PLGA	Poly Lactic-Co-Glycolic Acid
PR3	Proteinase 3
PTEN	Tensin Homolog Deleted on Chromosome 10
qPCR	Quantitative real-time Polymerase Chain Reaction
RANTES	Regulated Upon Activation, Normal T-cell Expressed and Secreted
RhoA	Ras Homolog Gene Family, Member A
RNA	Ribonucleic Acid
ROMK	Renal Outer Medullary Potassium Channel
ROS	Reactive Oxygen Species
rRNA	Ribosomal Ribonucleic Acid
SERPINE1	Serine Protease Inhibitor Protein 1
siRNA	Small Interfering Micro Ribonucleic Acid
SLE	Systemic Lupus Erythematosus
SPRY1	Sprouty Homolog 1
sRNA-seq	Small RNA Sequencing
TGF-β	Transforming Growth Factor Beta
TNF-α	Tumor Necrosis Factor Alpha
tRNA	Transfer Ribonucleic Acid
VSMCs	Vascular Smooth Muscle Cells
WNK	with no Lysine Kinase System
ZEB2	Zinc Finger E-Box-Binding Homeobox 2
